# High Hidden Burden of Diabetes Mellitus among Adults Aged 18 Years and Above in Urban Northwest Ethiopia

**DOI:** 10.1155/2020/9240398

**Published:** 2020-11-22

**Authors:** Haileab Fekadu Wolde, Terefe Derso, Gashaw Andargie Biks, Mezgebu Yitayal, Tadesse Awoke Ayele, Kassahun Alemu Gelaye, Getu Debalkie Demissie, Telake Azale, Bisrat Misganaw, Adane Kebede, Destaw Fetene Teshome, Endalkachew Dellie, Tsegaye Gebremedhin, Asmamaw Atnafu

**Affiliations:** ^1^Department of Epidemiology and Biostatistics, Institute of Public Health, College of Medicine and Health Sciences, University of Gondar, Gondar, Ethiopia; ^2^Department of Human Nutrition, Institute of Public Health, College of Medicine and Health Sciences, University of Gondar, Gondar, Ethiopia; ^3^Dabat Health and Demographic Surveillance System Research Centre, Institute of Public Health College of Medicine and Health Science, University of Gondar, Ethiopia; ^4^Department of Health Systems and Policy, Institute of Public Health, College of Medicine and Health Sciences, University of Gondar, Gondar, Ethiopia; ^5^Department of Health Promotion and Behavioral Sciences, Institute of Public Health, College of Medicine and Health Sciences, University of Gondar, Gondar, Ethiopia

## Abstract

**Background:**

Ethiopia is one of the sub-Saharan African countries with a rapidly increasing burden of diabetes mellitus (DM). There is limited updated information about the community-based burden of the disease and its associated factors in Ethiopia which is very crucial to plan effective prevention and control measures against the disease. This study is aimed at determining the burden of DM and its associated factors in urban northwest Ethiopia.

**Methods:**

A community-based cross-sectional study was conducted from April to May 2019 among residents aged ≥ 18 years in Gondar town and urban kebeles (lowest administrative units of the country) of Health and Demographic Surveillance System site (HDSS) in Dabat district. A multistage sampling technique was used to select 773 participants. World Health Organization (WHO) stepwise approach for noncommunicable disease surveillance was used to collect the data. Fasting blood glucose (FBS) ≥ 126 mg/dl was used to diagnose DM. Descriptive statistics were done to describe the variables of the study. Prevalence with its 95% confidence interval (CI) was estimated. Binary logistic regression model was fitted, variables with *p* value < 0.05 were considered to have a significant association with the outcome, and odds ratio (OR) was used to measure the strength of association.

**Result:**

Of the total participants, 6.34% (95% CI; 4.82, 8.29) were found to be diabetic. Of these, 40 (81.6%) were newly diagnosed. Besides, the prevalence of prediabetes was 9.31% (95% CI: 7.45, 11.58). Increased age (AOR = 1.06, 95% CI; 1.04, 1.09) and eating vegetables one to three days per week (AOR =0.29, 95% CI; 0.13, 0.65) were significantly associated with diabetes.

**Conclusion:**

The overall prevalence of DM is a bit higher than the national estimate, while the proportion of undiagnosed DM which can easily progress to disabling and life-threatening complications was alarmingly high. Age and frequency of eating vegetables per week were associated with diabetes. In light of this finding, future prevention and control measures against the diseases should consider the identified factors. There should also be improved access to screening services.

## 1. Background

Diabetes mellitus (DM) is a chronic metabolic disorder characterized by chronic hyperglycemia, and it is becoming an emerging public health problem because of its increasing prevalence, association with cardiovascular disease, and mortality [1, 2]. Globally, the prevalence of DM was 8.5% in 2016, and it is estimated to be one adult in ten will have diabetes in the world by 2035 [3]. Sub-Saharan Africa (SSA) countries are expected to experience the worldwide fastest increase in the number of people living with type 2 diabetes in the next two decades [4]. It is estimated that developing countries including Ethiopia will bear 77% of the global burden of the DM epidemic in the 21st century [5]. Globally, around 4.6 million deaths are attributed to DM annually, and it is one of the top ten causes of disability worldwide which undermines productivity and human development [6]. In SSA, the challenge posed by DM is even more overwhelming since diabetes will have to share scarce resources with infectious diseases and malnutrition [7]. The proportion of undiagnosed DM in Africa (66.7%) is almost two times higher than that of developed countries which is 37% [8]. This also contributes to the higher burden of morbidity and mortality at an early age in Africa.

Ethiopia as one of the developing countries has been showing changes that shift the lifestyle of the people towards urbanization, dietary changes, and reduced physical activity, particularly in recent decades. These rapid changes have led to the emergence of noncommunicable diseases (NCDs) such as diabetes mellitus [9]. In 2017, the projected national diabetes prevalence among individuals aged 20-79 was estimated to be 5.2% in Ethiopia, and there are 2.6 million DM cases in the country [10, 11].

Different factors that are associated with DM have been identified by previous studies. This include age [12–15], educational status [13], residence [12, 16], sex [12], and family history of DM [2, 14, 15, 17], hypertension [2, 13, 16, 18], central obesity [2, 16, 18], total cholesterol [18], and physical activity [14, 15].

The economic burden of DM and the handicaps because of its complications can easily plunge families into poverty [12, 19]. This burden becomes much higher if the disease is not diagnosed and treated early because undiagnosed DM may lead to complications like retinopathy, nephropathy, and neuropathy [20] that may end up with irreversible disabilities or death. Globally, the proportion of end-stage renal diseases attributable to diabetes alone ranges from 12–55%, and its incidence is ten times higher among diabetic patients [3]. Diabetic retinopathy also affects one in three people with diabetes and remains the leading cause of blindness in working-age adults [21]. Despite the increase in the prevalence of DM, its complications, and the negative impact on the health system, few community-based studies are available in Ethiopia. Knowing the prevalence of DM and its associated factors provides information for policymakers to plan prevention, early diagnosis, and intervention methods. However, low attention is given to community-level screening, early prevention, and control of DM. Therefore, the aim of this study was to assess the prevalence of DM and its associated factors among adults above the age of 18 years in urban northwest Ethiopia.

## 2. Methods

### 2.1. Study Design and Setting

Community-based cross-sectional study design was conducted from April to May 2019 among the adult population aged 18 years and above living in Gondar town and urban kebeles (lowest administrative unit of the county) of Health and Demographic Surveillance System site (HDSSs) in Dabat district. Gondar town is found in North Gondar Zone, Amhara Regional State of Ethiopia, and is located at 750 km from Addis Ababa to the northwest. Based on the 2007 Central Statistical Agency (CSA) report of Ethiopia, the town had a total population of 400,000. From these, 198,120 are men and 201,880 are women. Administratively, the town is divided into 12 administrative areas (subcities) and 23 kebeles (the smallest administrative units in Ethiopia). The town has 48 health institutions; these are 1 comprehensive referral hospital, 8 health centers, 1 private general hospital, 15 specialty clinics, 15 medium clinics, and 8 primary clinics. Dabat HDSS site is located in Dabat District, northwest Ethiopia. The site was established in 1996. It covers a total of 13 kebeles, (9 rural and 4 urban). The kebeles in the surveillance site were selected randomly, by taking all ecological zones (high land, middle land, and lowland) into account, and this study includes households in all the urban kebeles.

### 2.2. Population, Sampling Procedure, and Sample Size Determination

The source population for this study was all adults above the age of 18 years living for ≥6 months in Gondar and Dabat district. The study population was those adults in the selected kebeles and who were available during the data collection period. Pregnant mothers during the postpartum period, individuals who were taking any drug with possible impact on glucose metabolism (i.e., steroids, B-blockers, and thiazide diuretics), and sick individuals during the data collection period were excluded from the study. The sample size for the prevalence was determined using single population proportion formula by assuming 95% CI, 0.05 margins of error, 10% nonresponse rate, and 2 design effect and proportion of DM to be 5.1% [14] and found a sample size of 163. Moreover, the sample size for the analytic component was determined using the power approach, and the final sample size was found to be 805.

The sample was selected using multistage sampling. At the first stage, two study sites (Gondar town, and Dabat HDSS) were selected; at the second stage, 6 kebeles (smallest administrative units of the country) from Gondar town and 4 urban kebeles of Dabat HDSS were randomly selected; at the third stage, the sample size was distributed for each kebeles, and systematic random sampling technique was used to select households in each kebele. In the presence of more than one eligible individual in a single household, a lottery method was used to select one.

### 2.3. Variables and Data Collection Procedure

The dependent variable for this study was diabetes mellitus. Blood glucose was measured using CareSenseN glucometer, and DM was diagnosed based on WHO guideline which was fasting blood sugar (FBS) ≥ 126 mg/dl [22] or self-report of previous diagnosis of DM by a health professional or currently taking treatment for diabetes. The first group of factors assessed was sociodemographic characteristics. This includes age, sex, educational status, occupation, marital status, wealth index, and family history of DM and HTN. The second was behavioral factors, and this includes current smoking and alcohol consumption status, number of sitting hours per day, frequency of eating vegetables, fruit and fatty meal, type of oil used, and physical activity. A person is said to be physically active if he/she at least do moderate physical activities like carrying light loads for at least 10 minutes continuously or cycling, swimming, and volleyball for at least 10 minutes continuously. The third group of characteristics assessed was physical measurements. This includes waist circumference, blood pressure, weight, and height. Blood pressure was measured three times in a sitting position using a standard mercury sphygmomanometer BP cuff with the appropriate cuff size that covers two-thirds of the upper arm after the participant rested for at least five minutes and no smoking or caffeine 30 minutes before measurement. The second and the third measurements were taken five-to-ten minutes after the first and the second measurement, respectively. Finally, the average of the three BP measurements was calculated to determine the BP status of the participant. An individual was diagnosed as hypertensive if systolic blood pressure (SBP) is ≥140 mg/dl or diastolic blood pressure (DBP) is ≥90 mg/dl or previous diagnosis of hypertension or current use of antihypertensive drug [23]. Waist circumference (WC) was measured at an approximate midpoint between the lower margin of the lowest palpable rib and top of iliac crest using flexible plastic tape without heavy outdoor closing. WC of ≥94 cm for males and ≥80 cm for females was considered as high-risk WC [24]. Weight and height to calculate BMI was taken using calibrated equipment, and BMI was calculated by dividing weight in kg by height in meters square. BMI < 18.5 kg/m^2^ was considered as underweight 18.5-24.9 kg/m^2^ as normal, 25-29.9 kg/m^2^ overweight, and ≥30 kg/m^2^ as obese [25].

The data was collected using the World Health Organization (WHO) stepwise approach for noncommunicable disease surveillance which contains three steps [22]. In the first step, data on sociodemographic and behavioral factors was collected using a pretested semistructured interviewer-administered questioner which was prepared in the local language (Amharic) as per the recommendation by WHO stepwise approach. Questions related to alcohol use and smoking were modified to reflect the local context of Ethiopia. The second step was for physical measurements, and the third step was for biochemical measurement (blood glucose). Study participants who do not eat any food during the first contact were directly measured for fasting blood sugar. However, participants who eat food during the first contact were asked to fast overnight, and FBS was measured on the next day.

### 2.4. Data Processing and Analysis

The data were entered and cleaned using Epi-Info version 7 and analyzed using STATA version 14. Data cleaning and coding were made. Descriptive statistics in the form of means for continuous variables and percentages for categorical variables were made. Then, the findings were presented by tables and texts. Variables with 0.2 *p* values in the bivariable binary logistic regression analysis were fitted in the multivariable model. Adjusted odds ratio (AOR) with a 95% confidence interval (CI) and *p* value < 0.05 in the multivariable model were used to declare a significant association with the outcome. The goodness of fitness of the model was checked by Hosmer and Lemeshow test (*p* value = 0.555).

## 3. Result

### 3.1. Sociodemographic Characteristics of Participants

A total of 773 participants were involved in this study with a response rate of 96%. The median age of the participants was 33 (IQR = 26 − 48). The majority of the participants 477 (61.7%) were female, and 481 (62.2%) had at least secondary education. Of the participants, 334 (43.2%) were unemployed, and 408 (62.3%) were married. Besides, 308 (39.12%) were in the rich or richest wealth group, and only 57 (7.37%) had a family history of DM (Table 1).

### 3.2. Behavioral Characteristics and Physical Measurements of Participants

Of the total participants, more than two-third eat vegetables at least one day in a week and more than half 438 (56.7%) did not eat any fruits in a week. Besides, one-fourth of the participants did not eat fatty meals in a week and majority of 538 (69.9%) used the cruddy oil. Of all the respondents, only 15 (1.94%) were current smokers and majority of 476 (61.6%) drink alcohol in the last 30 days. Moreover, 502 (64.9%) of the participants had at least moderate physical activity on a typical day. Out of the total study participants, majority of 482 (62.4%) had normal waist circumference (Table 2).

### 3.3. Prevalence of DM among the Study Participants

A total of 49 cases of DM was found in this study with an overall prevalence of 6.34% (95% CI; 4.82, 8.29). The prevalence among male participants was 7.43% (95% CI: 4.71, 11.04), and the prevalence among female participants was 5.66% (95% CI: 3.76, 8.13). Of the total cases of diabetes, 40 (81.6%) were previously undiagnosed cases. Moreover, 72 of the participants were found to be prediabetic with a prevalence of 9.31% (95% CI: 7.45, 11.58).

### 3.4. Factors Associated with DM

From the multivariable logistic regression analysis, age and frequency of eating vegetables per week were found to be significantly associated with DM. For a one year increase in age, the odds of having DM is increased by 6% (AOR = 1.06, 95% CI; 1.04, 1.09). The odds of having DM was decreased by 71% among participants who eat vegetables one to three days per week compared to participants who did not eat (AOR = 0.29, 95% CI; 0.13, 0.65) (Table 3).

## 4. Discussion

This study mainly assessed the prevalence of DM and its associated factors among adults in urban northwest Ethiopia and found age and frequency of eating vegetables per week to be significantly associated with DM.

In this study, the prevalence of DM was found to be 6.34%. The result was consistent with a study conducted in Mizan-Aman, Ethiopia (6.5%) [18], and a systematic review and meta-analysis done in Ghana (6.46%) [15]. However, the result was somewhat higher than the estimated national prevalence (5.2%) reported by IDF in 2017 [11], and it was much higher than the findings from rural settings of Ethiopia which reported prevalence between 1.9 and 2.1% [14, 16, 26] and rural Sudan (2.6%) [27]. This could be due to the difference in study settings because the current study was conducted only among urban residents which may increase the prevalence. After all, people living in urban areas are more exposed to the risk factors for the conditions like decreased physical activity and unhealthy diet than the rural, and this increases the risk of developing noncommunicable diseases like DM [28]. This finding is one of the indicators that the disease is becoming an emerging public health problem especially in urban areas than rural. Besides, the prevalence of DM in this study was a bit higher than findings from different urban settings of Ethiopia which reported prevalence between 2.6% and 5.1% [2, 14, 16, 29]. This difference could be attributed to the difference in time between the studies because there is still an increasing trend of urbanization and change in lifestyles which can contribute to the higher burden of the disease currently. This finding can also be considered alarming for the increasing trend of the diseases from time to time. On the other hand, the prevalence from this study was lower than the studies from Bangladesh (9.7%) [30], Saudi Arabia (12.1%) [31], China (10.4%) [32], Kenya (14%) [33], and India (17.1%) [17]. This might be due to the difference in sociodemographic characteristics and lifestyles between the countries. Another reason could be due to the difference in characteristics of the study participants because the study from Kenya was done only among hypertensive adults, and this may increase the risk of DM as evidenced by different studies [2, 30].

Another surprising result from the current study was 81.6% of the DM cases were newly diagnosed. This result was comparable with the finding from Mizan-Aman, Ethiopia (88.5%). However, it was higher than the findings from other studies reported from other sites in Ethiopia [14, 16, 26] and elsewhere [34, 35]. This could be attributed to the low awareness of the community and the health professionals about the disease. It may also be due to the poor health-seeking behavior of the community unless the health problem shows severe signs and symptoms or they perceive that the disease is severe [36]. This result is alarming for the Ministry of Health, the community, and all the concerned bodies because it indicates that there is much proportion of undiagnosed diabetes in the community, and these individuals seek medical care after they develop severe and disabling complications of the disease which can easily be prevented if the disease was diagnosed early. This result can also be used as a baseline to evaluate the progression of prevention and control measures against the disease.

In this study, higher odds of having DM was found as age increases. This was supported by other studies [14, 31, 34, 37, 38]. This can be due to the decline in lean body mass and increased body fat, particularly visceral adiposity that accompanies increasing age and this leads to insulin resistance [39]. Moreover, increased age is also associated with decreased Islet cell function [40]. Therefore, the screening services for DM should give special attention to individuals with increased age.

The odds of having DM was lower among participants who eat vegetables one to three days per week compared to those who did not eat. This was supported by a review [41] which sowed the protective effects of eating vegetables and fruits from chronic diseases like DM. This may be due to the fiber found in vegetables since vegetable fiber support lowering of serum cholesterol and slowing the release of sugar into the bloodstream, thus decreases the fasting blood glucose level between 6 and 39% [41, 42]. In a country like Ethiopia with a large number of vegetable productions, this can be one of the cost-effective measures for prevention of the diseases.

Though it is not statistically significant in this study, low physical activity is proven to increase the odds of having DM by studies done elsewhere [14, 15]. The possible reason for this could be the effect of physical activity in improving insulin action by maintaining skeletal muscle mass. Skeletal muscle mass is the major site of glucose disposal in the human body, so physical activity improves insulin action primarily by maintaining skeletal muscle mass and improving insulin sensitivity [43]. Physical exercises can also decrease adipose tissue mass, especially in visceral organs even if weight loss does not occur. This reduction in adipose tissue mass can also be accompanied by reductions in cardiometabolic risk factors including insulin resistance [43].

The clinical importance of this study could include allows us to easily identify individuals at risk of DM and to provide early diagnosis and intervention. It also has a huge public health benefit because it gives information for policymakers to design an effective strategy towards the prevention of the diseases. This in turn will help to decrease the economic loss because of DM and the handicaps related to its complications that can easily lead families of the patient and the country into poverty. The limitation of this study was it could not establish cause and effect relationships because of the cross-sectional nature of the study design, and there could also be some measurement errors.

## 5. Conclusion

The overall prevalence of DM is a bit higher than the national estimate. However, the proportion of undiagnosed DM which can easily progress to disabling and life-threatening complications was alarmingly high. Age and frequency of eating vegetables per week were associated with diabetes. In light of this finding, future prevention and control measures against the diseases should consider the identified factors. There should also be improved access to screening services.

## Figures and Tables

**Table 1:** Sociodemographic characteristics of adults aged 18 years and above in urban northwest Ethiopia, 2019.

Variable	Blood sugar status			Total—*n* (%)
	Normal—*n* (%)	Prediabetes—*n* (%)	Diabetes—*n* (%)	
Age				
18-23	121 (18.6)	7 (9.7)	3 (6.1)	131 (17.0)
24-28	136 (20.9)	7 (9.7)	1 (2.0)	144 (18.6)
29-36	153 (23.5)	16 (22.2)	5 (10.2)	174 (22.5)
37-50	142 (21.8)	13 (18.1)	12 (24.5)	167 (21.6)
>50	100 (15.3)	29 (40.3)	28 (57.1)	157 (20.3)
Sex				
Male	244 (37.4)	30 (41.7)	22 (44.9)	296 (38.3)
Female	408 (62.6)	42 (58.3)	27 (55.1)	477 (61.7)
Education				
Uneducated	81 (12.4)	19 (26.4)	10 (20.4)	110 (14.2)
Able to read and write	44 (6.8)	13 (18.1)	6 (12.2)	63 (8.2)
1-4	15 (2.3)	2 (2.8)	0 (0.0)	17 (2.2)
5-8	87 (13.3)	7 (9.7)	8 (16.3)	102 (13.2)
9-10	117 (17.9)	9 (12.5)	6 (12.2)	132 (17.1)
11-12	67 (10.3)	6 (8.3)	5 (10.2)	78 (10.1)
Diploma and above	241 (37.0)	16 (22.2)	14 (28.6)	271 (35.1)
Occupation				
Unemployed	286 (43.9)	31 (43.1)	17 (34.7)	334 (43.2)
Government employed	203 (31.1)	15 (20.8)	9 (18.4)	227 (29.4)
Private	144 (22.1)	20 (27.8)	12 (24.5)	176 (22.8)
Retired	19 (2.9)	6 (8.3)	11 (22.6)	36 (4.7)
Religion				
Orthodox	578 (88.7)	66 (93.0)	44 (89.8)	688 (89.1)
Muslim	70 (10.7)	5 (7.0)	5 (10.2)	80 (10.4)
Protestant	4 (0.6)	0 (0.0)	0.(0.0)	4 (0.5)
Marital status				
Single	189 (29.0)	12 (16.9)	5 (10.2)	206 (26.7)
Married	406 (62.4)	43 (60.6)	31 (63.3)	480 (62.3)
Divorced	30 (4.6)	12 (16.9)	1 (2.0)	43 (5.6)
Widowed	26 (4.0)	4 (5.6)	12 (24.5)	42 (5.4)
Wealth index				
Poorest	139 (21.3)	6 (8.3)	7 (14.3)	152 (19.7)
Poor	132 (20.3)	18 (25.0)	7 (14.3)	157 (20.3)
Medium	111 (17.0)	32 (44.4)	13 (26.5)	156 (20.2)
Rich	133 (20.4)	8 (11.1)	13 (26.5)	154 (19.9)
Richest	137 (21.0)	8 (11.1)	9 (18.4)	154 (19.9)
Family history of DM				
Yes	47 (7.2)	3 (4.2)	7 (14.3)	57 (7.4)
No	605 (92.8)	69 (95.8)	42 (85.7)	716 (92.6)
Family history or HTN				
Yes	96 (14.7)	8 (11.1)	7 (14.3)	111 (14.4)
No	556 (85.3)	64 (88.9)	42 (85.7)	662 (85.6)

DM: diabetes mellitus; HTN: hypertension.

**Table 2:** Behavioral characteristics of adults aged 18 years and above in urban northwest Ethiopia, 2019.

Variable	Blood sugar status			Total—*n* (%)
	Normal—*n* (%)	Prediabetes—*n* (%)	Diabetes—*n* (%)	
Frequency of eating vegetables per week				
No eating	182 (27.9)	37 (51.4)	23 (46.9)	242 (31.3)
1-3 times	359 (55.1)	23 (31.9)	15 (30.6)	397 (51.4)
4-7 times	111 (17.0)	12 (16.7)	11 (22.5)	134 (17.3)
Frequency of eating fruit per week				
No eating	364 (55.8)	44 (61.1)	30 (61.2)	438 (56.7)
1-3 times	226 (34.7)	24 (33.3)	15 (30.6)	265 (34.3)
4-7 times	62 (9.5)	4 (5.6)	4 (8.2)	70 (9.1)
Frequency of eating fatty meal per week				
No eating	496 (76.1)	48 (66.7)	35 (71.4)	579 (74.9)
1-3 times	108 (16.6)	13 (18.1)	8 (16.3)	129 (16.7)
4-7 times	48 (7.4)	11 (15.3)	6 (12.2)	65 (8.4)
Type of oil				
Cruddy oil	456 (69.9)	51 (70.8)	31 (63.3)	538 (69.6)
Liquid oil	196 (30.1)	21 (29.1)	18 (36.7)	235 (30.4)
Current smoking				
Yes	14 (2.2)	0 (0.0)	1 (2.0)	15 (1.94)
No	638 (97.8)	72 (100.0)	48 (98)	758 (98.1)
Drink alcohol in the last 30 days				
Yes	261 (40.0)	25 (34.7)	11 (22.4)	297 (38.4)
No	391 (60.0)	47 (65.3)	38 (77.6)	476 (61.6)
Number of hours sitting in typical day				
1-5	588 (90.3)	68 (94.4)	42 (85.7)	698 (90.4)
6-12	63 (9.7)	4 (5.6)	7 (14.3)	74 (9.6)
At least moderate physical activity				
Yes	443 (67.9)	36 (50.0)	23 (46.9)	502 (64.9)
No	209 (32.1)	36 (50.0)	26 (53.1)	271 (35.1)
Waist circumference				
Normal	419 (64.3)	42 (58.3)	21 (42.9)	482 (62.4)
High risk	233 (35.7)	30 (41.7)	28 (57.1)	291 (37.6)
BP status				
<140/90 mmHg	581 (89.1)	66 (91.7)	36 (73.5)	683 (88.4)
≥140/90 mmHg	71 (10.9)	6 (8.3)	13 (26.5)	90 (11.6)

BP: blood pressure; High-risk waist circumference: ≥94 cm for male and ≥80 cm for female.

**Table 3:** Binary logistic regression output for the factors associated with DM among adults aged 18 years and above in urban northwest Ethiopia, 2019.

Variable	DM		Crude OR (95% CI)	Adjusted OR (95% CI)
	Yes	No		
Age			1.06 (1.04, 1.08)	1.06 (1.04, 1.09)^∗∗^
Sex				
Male	22	274	1	1
Female	27	450	0.75 (0.42, 1.34)	0.76 (0.34, 1.70)
Educational status				
Uneducated	16	157	1.87 (0.89, 3.94)	0.35 (0.11, 1.14)
Primary	8	111	1.32 (0.54, 3.24)	0.83 (0.25, 2.74)
Secondary	11	199	1.01 (0.45, 2.28)	1.00 (0.36, 2.81)
Diploma and above	14	257	1	1
Occupation				
Government employed	9	218	1	1
Unemployed	17	317	1.30 (0.57, 2.97)	1.49 (0.49, 4.52)
Private job	12	164	1.77 (0.73, 4.31)	1.61 (0.52, 4.95)
Retired	11	25	10.66 (4.03, 28.21)	2.04 (0.64, 6.53)
Wealth index				
Poor	14	295	1	1
Medium	13	143	1.92 (0.88, 4.18)	1.45 (0.52, 4.05)
Rich	22	286	1.62 (0.81, 3.23)	1.64 (0.61, 4.41)
Family history DM				
No	42	674	1	1
Yes	7	50	2.25 (0.96, 5.26)	2.15 (0.78, 5.95)
Frequency of eating vegetables per week				
No eating	23	219	1	1
1-3 days	15	382	0.37 (0.19, 0.73)	0.29 (0.13, 0.65)∗
4-7 days	11	123	0.85 (0.40, 1.81)	0.51 (0.19, 1.33)
Frequency of eating fatty meal per week				
No eating	35	544	1	1
1-3 days	8	121	1.03 (0.47, 2.27)	1.22 (0.49, 3.08)
4-7 days	6	59	1.58 (0.64, 3.91)	1.42 (0.51, 3.96)
Physical activity				
At least moderate physical activity	22	471	1	1
No physical activity	27	252	2.29 (1.28, 4.11)	1.70 (0.84, 3.44)
Current smoking habit				
Smoker	1	14	1.06 (0.14, 8.20)	0.96 (0.09, 10.45)
Nonsmoker	48	710	1	1
Waist circumference				
Normal	21	461	1	1
High risk	28	263	2.00 (1.12, 3.60)	1.72 (0.82, 3.60)
Hypertension				
No	36	647	1	1
Yes	13	77	3.03 (1.54, 5.97)	1.35 (0.61, 2.99)

AOR: adjusted odds ratio; COR: crude odds ratio; CI: confidence interval; DM: diabetes mellitus; High-risk waist circumference: ≥94 cm for male and ≥80 cm for female; ^∗^*p* value < 0.01; ^∗∗^<0.001.

## Data Availability

Data will be available from the corresponding author upon request.

## Abbreviations

AOR: Adjusted odds ratio
BMI: Body mass index
BP: Blood pressure
CI: Confidence interval
COR: Crude odds ratio
CSA: Central statistical agency
DBP: Diastolic blood pressure
DM: Diabetes mellitus
FBS: Fasting blood sugar
HDSS: Health and demographic surveillance system site
IDF: International Diabetes Federation
IQR: Interquartile range
NCD: Noncommunicable disease
SBP: Systolic blood pressure
WHO: World Health Organization.
